# Aging-associated changes of optical coherence tomography-measured ganglion cell-related retinal layer thickness and visual sensitivity in normal Japanese

**DOI:** 10.1007/s10384-024-01049-3

**Published:** 2024-03-18

**Authors:** Aiko Iwase, Tomomi Higashide, Makoto Fujii, Yuko Ohno, Yuki Tanaka, Tsutomu Kikawa, Makoto Araie

**Affiliations:** 1 Tajimi Iwase Eye Clinic, 3-101-1, Honmachi, Tajimi, Gifu Prefecture 507-0033 Japan; 2 Department of Ophthalmology, Kanazawa University Graduate School of Medical Sciences, Kanazawa, Japan; 3 Division of Health and Sciences, Osaka University Graduate School of Medicine, Osaka, Japan; 4 Santen Pharmaceutical, Tokyo, Japan; 5 Topcon Corporation, Tokyo, Japan; 6 Kanto Central Hospital of the Mutual Aid Association of Public School Teachers, Tokyo, Japan

**Keywords:** Circumpapillary retinal nerve fiber layer thicknes, Macular ganglion cell-inner plexiform layer thickness, Macular ganglion-cell complex thickness, Aging-associated decline rate, Normal healthy eyes

## Abstract

**Purpose:**

To report aging-associated change rates in circumpapillary retinal nerve fiber layer thickness (cpRNFLT) and macular ganglion cell-inner plexiform layer and complex thickness (MGCIPLT, MGCCT) in normal Japanese eyes and to compare the data in linear scaled visual field (VF) sensitivity of central 4 points of Humphrey Field Analyzer (HFA) 24-2 test (VF_4TestPoints_) to that in MGCIPLT in four 0.6-mm-diameter circles corresponding to the four central points of HFA 24-2 adjusted for retinal ganglion cell displacement (GCIPLT_4TestPoints_).

**Study design:**

Prospective observational study

**Methods:**

HFA 24-2 tests and spectral-domain optical coherence tomography (SD-OCT) measurements of cpRNFLT, MGCIPLT, MGCCT and GCIPLT_4TestPoints_ were performed every 3 months for 3 years in 73 eyes of 37 healthy Japanese with mean age of 50.4 years. The time changes of SD-OCT-measured parameters and VF_4TestPoints_ were analyzed using a linear mixed model.

**Results:**

The aging-associated change rates were -0.064 μm/year for MGCIPLT and and -0.095 for MGCCT (P=0.020 and 0.017), but could not be detected for cpRNFLT. They accelerated with aging at -0.009μm/year/year of age for MGCIPLT (P<0.001), at 0.011 for MGCCT (P<0.001) and at 0.013 for cpRNFLT(0.031). The aging-associated decline of -82.1 [1/Lambert]/year of VF_4TestPoints_ corresponded to -0.095 μm/year of GCIPLT_4TestPoints_.

**Conclusion:**

We report that aging-associated change rates of cpRNFLT, MGCIPLT and MGCCT in normal Japanese eyes were found to be significantly accelerated along with aging. Relationship between VF sensitivity decline rates and SD-OCT measured GCIPLT decline rates during physiological aging in the corresponding parafoveal retinal areas are also documented.

**Supplementary Information:**

The online version contains supplementary material available at 10.1007/s10384-024-01049-3.

## Introduction

Histologic studies in human eyes have estimated that about 7000 retinal ganglion cells (RGCs) are lost during normal aging [1–5]. In accordance with these findings, cross-sectional and longitudinal studies in normal human eyes show that visual field (VF) sensitivity measured using standard automated perimetry (SAP) [6–10] and the thickness of the RGC-related retinal layers (RGC-RRLT) determined by spectral-domain optical coherence tomography (SD-OCT) showed physiological aging-associated decline [11–22]. To estimate disease-caused time-related changes of RGC-RRLT, it is mandatory to deduct the physiological aging-associated changes from the measured values.

It is well known that there are ethnical differences in the RGC-RRLT measurement results in normal subjects [14, 15, 21], and that RGC-RRLTs measurement results strongly depend on the SD-OCT instruments used [23–27]. Although there are reports of aging-associated decline rates of RGC-RRLT in other ethnical populations [17–22], there is a paucity of information on the SD-OCT instrument-specific aging-associated decline rates of RGC-RRLT in healthy Japanese. Both glaucoma and the physiological aging process reduce the RGC-RRLT and VF sensitivity [6–22]. In glaucoma eyes, the decline rates of SD-OCT measured RGC-RRLT and VF sensitivity in the corresponding retinal area were compared, and in glaucoma eyes with early or pre-perimetric damage, changes in the circumpapillary retinal nerve fiber layer thickness (cpRNFLT) are reported to generally precede glaucomatous declines in the VF sensitivity detected using the Humphrey Field Analyzer 24-2 Swedish Interactive Thresholding Algorithm (SITA) program (HFA 24-2, Carl Zeiss Meditec) [28–31]. However, a comparison of aging-associated declines in SD-OCT-measured RGC-RRLT and VF sensitivity in the corresponding retinal areas are not reported in the same subjects’ eyes.

The purpose of the current study was two-fold. 1) To prospectively measure the longitudinal time changes in the cpRNFLT, macular ganglion cell-inner plexiform layer thickness (MGCIPLT) and ganglion cell complex thickness (MGCCT) and report the aging-associated decline rate of cpRNFLT, MGCIPLT and MGCCT in normal Japanese, and 2) to compare the aging-associated changes of the MGCIPLT to VF sensitivity obtained in the corresponding retinal area in the same eye.

## Methods

Subjects. Self-reported healthy Japanese individuals were recruited at the Tajimi Eye Clinic (Tajimi, Gifu, Japan). After subjects were screened verbally and medical histories recorded, an ocular examination was performed that included measurements of the uncorrected and autorefraction-corrected visual acuity (VA) with a Landolt chart at 5 meters and the corneal curvature using an autorefractometer (KR-800A, Topcon). In addition, the central corneal thickness and axial length (AXL) were measured, respectively, using a specular microscope (SP-3000P, Topcon) and the IOLMaster (Carl Zeiss Meditec). The VFs were examined using SAP (HFA 24-2 SITA program, Carl Zeiss Meditec). The VF examination was repeated whenever it was considered unreliable or outside the normal limits. The SD-OCT examination was followed by dilated optic disc stereo photography and fundus photography, dilated funduscopy, slit-lamp biomicroscopy, and intraocular pressure (IOP) measurements by Goldmann applanation tonometry. A pair of sequential stereoscopic optic nerve head photographs at a parallax of about 8 degrees (30-degree angle of view) and non-stereoscopic fundus photographs (45-degree angle of view) was obtained using a digital fundus camera (TRC-NW7, Topcon) after pupillary dilation with 1.0% tropicamide. All ocular examinations were performed bilaterally.

The inclusion criteria were age between 20 and 75 years; normal eye examinations without any clinically significant cataract, ocular media, vitreoretinal, or choroidal abnormalities; IOP of 21 mmHg or lower; best-corrected decimal VA of 1.0 or higher; spherical refraction of ± 6 diopters (D) or less; astigmatism of 2 D or less; AXL of 26 mm or less; no previous ocular surgery; normal VF test results with the glaucoma hemifield test, and mean deviation and pattern standard deviation within normal limits. Subjects were excluded if the VF results were unreliable based on the perimetrist’s notes and reliability indices with fixation loss and false positive rates of over 20% and over 15%, respectively; the optic disc stereo photographs were of insufficient quality; or the OCT images were of insufficient quality (typically truncated B-scans and scans with a manufacturer-authorized image quality score of 30 or lower). After enrollment, routine ophthalmic examinations, SD-OCT, and VF measurements were prospectively performed every 3 months for 3 years.

The Review Board and Ethics Committee of Gifu Prefecture Medical Association approved the study (reference number, 25-1-001), which adhered to the tenets of the Declaration of Helsinki. The study was registered in the University Hospital Medical Information Network Clinical Trial Registry (UMIN-000012412). Each subject provided written informed consent after receiving a full explanation of the study protocol.

SD-OCT. SD-OCT data sets were obtained using a 3D-OCT 2000 (Topcon) with the horizontal 3-dimensional (3D) scan protocol in which data were obtained from 6.0 × 6.0-mm-square areas (512 A-scans × 128 frames) centered on the disc and with the vertical 3D scan protocol in which data were obtained from a 7.0 × 7.0-mm-square area (512 A-scans × 128 frames) centered on the fovea over a period of about 1.5 seconds for each scan. The points on the OCT image sensor plane corresponding to the measurement targets were determined according to the manufacturer-provided calculations; these calculated the relationship between the points in the posterior fundus of each subject eye and those on the SD-OCT image obtained from each eye based on refractive error, corneal radius, AXL of each subject eye and Gullstrand schematic eye [32] (Supplement). The data obtained in the presence of eye movements were discarded and the examination was repeated. Images also were excluded whenever they were affected by involuntary blinking or saccades, indicated by breaks, shifting of the vessels, or the presence of a straight line across the fundus OCT image, or had an image quality score of 30 or less. OCT measurements were repeated 3 times within several-second intervals, and the image with the best quality was used. The disc barycenter was determined using the raster-scan data, and the fovea identified in the OCT image as the thinnest pixel between the inner limiting membrane and the photoreceptor inner/outer segment junction (ellipsoid zone) adjacent to the fixation point. The RNFL and GCIPL (GCC) were segmented automatically in all B-scan images [33]. To minimize variability due to misplacement of the measurement location and/or segmentation error, an experienced researcher (T.K.) checked the locations of the disc barycenter and fovea and all layer segmentation in all images. The thickness of RNFL was measured along the 3.4-mm-diameter circle centered on the disc center (cpRNFLT), and that of macular GCIPL (GCC) {MGCIPLT(MGCCT)} was obtained from a 6.0 × 6.0-mm-square area centered on the fovea.

The MGCIPLT in a 0.6-mm-diameter circular retinal area (corresponding to about 2 degrees of visual angle) corresponding to each of the four central test points of the HFA 24-2 and adjusted for the RGC displacement [34] was obtained and the mean of these 4 measurement results were obtained (GCIPLT_4TestPoints_). The diameter of the retinal area (2 degrees) was like the grid size of the HFA 10-2 test program and roughly twice as large as the size-III stimulus point movements during fixation, such as drift.

## Data analysis

Since no previous studies report the aging-associated change rates of cpRNFLT, MGCCT or MGCIPLT in normal Japanese eyes, the variation of the physiological aging-associated change rates of these SD-OCT parameters in Japanese needed for sample size calculation was unknown. When the study was planned, four papers already reported the physiological aging-related changes of cpRNFLT and MGCCT or MGCIPLT in normal subjects using the cohorts mainly consisting of Caucasians [17–20]. In these studies, the mean number of normal subjects included was 35, follow-up period was 36.3 months, follow-up interval was 4.4 months and there were 8 visits during the follow-up. Based on these studies, we planned to enroll 38 normal subjects who were to be examined at 3 month-intervals for 36 months (12 visits) and assumed that, in that way reliable data of physiological aging-associated changes of CpRNFLT, MGCCT or MGCIPLT in normal Japanese could be collected.

The results are expressed as the mean (standard deviation). The effects of aging (duration or time lapse from the baseline measurement) on the cpRNFLT, MGCIPLT, MGCCT and GCIPLT_4TestPoints_ were analyzed, using the time changes of the cpRNFLT, MGCIPLT, MGCCT and GCIPLT_4TestPoints_ values during follow-up as dependent variables and using the linear mixed model, which considers correlations between the paired eyes and measurement values from the same eyes. The explanatory variables were duration (time lapse from the baseline measurement), baseline age, baseline cpRNFLT, MGCIPLT, MGCCT or GCIPLT_4TestPoints_, AXL [12, 16] and gender [35–37], image quality index [38, 39].

The decibel values for VF sensitivity in test points of the HFA 24-2 test program were anti-logged to obtain the sensitivity in the linear scale (1/Lambert=10^(0.1×dB)^, linear sensitivity) [40, 41]. Effects of aging (duration or time lapse from the baseline measurement) on the mean linear VF sensitivity over the whole area of the HFA-24-2 (VF_mean_) and the central 4 test points of the HFA 24-2 (VF_4TestPoints_) were analyzed, using the time changes of the VF_mean  _or VF_4TestPoints_ values during follow-up as a dependent variable and a linear mixed model, which considers correlations between the paired eyes and measurement values from the same eyes. The explanatory variables were duration (time lapse from the baseline measurement), baseline age, baseline VF_mean_ and VF_4TestPoint_ and AXL [10, 42, 43].

STATA software (version 17.0, Stata Corp) and ^The^ JMP^®^ Pro 13 software (SAS Institute Inc.) were used for analyses and contribution of an explanatory variable with P<0.050 was adopted as to be significant.

## Results

A total of 76 eyes of 38 normal subjects were enrolled; one eye of one subject was excluded because of development of epiretinal membrane and vitreoretinal traction during follow-up, and two eyes of one subject were excluded because reliable SD-OCT measurements could not always be obtained mainly due to saccades and blinking. The demographics of the remaining 73 eyes of 37 subjects are shown in Table 1. During the 3-year prospective follow-up, ocular transparent media including lens showed no changes on biomicroscopic examination.

**Table 1:** Summary of subjects’ characteristics

Parameter	Mean (standard deviation)
Number of subjects (eyes; right/left)	37 (73; 36/37)
Age (years)	50.4 (12.3)
Axial length (mm)	24.3 (1.0)
Spherical equivalent refraction (diopters)	− 1.55 (2.02)
Best-corrected decimal visual acuity	1.5 (0.9 ~ 1.5)*
Central corneal thickness (μm)	515 (29)
Corneal curvature (mm)	7.75 (0.28)
Intraocular pressure (mmHg)	13.6 (0.8)
Disc area (mm^2^)	2.21 (0.37)
CpRNFLT (μm)	107.1 (8.6)
MGCIPLT (μm)	67.2 (4.2)
MGCCT (μm)	105.9 (6.8)
VF_mean_ (1/Lambert)	1223.2 (294.8)
VF_4TestPoints_ (1/Lambert)	2125.5 (498.7)
GCIPLT_4TestPoints_ (μm)	88.1 (6.0)

The data are expressed as the mean (standard deviation). VF_mean_, mean linear visual field sensitivity of the whole test points of the HFA 24-2; VF_4TestPoints_, mean linear visual field sensitivity of the central 4 test points of the HFA 24-2; cpRNFLT, circumpapillary retinal nerve fiber layer thickness; MGCIPLT (MGCCT), mean maculalr ganglion cell-inner plexiform layer thickness (ganglion cell complex thicknesses); GCIPLT_4TestPoints_, mean ganglion cell-inner plexiform layer thicknesses in a circular retinal area with a diameter of 0.6 mm (approximately 2 degrees of the visual angle) corresponding to the four central test points of the HFA 24-2, adjusted for RGC displacement according to Drasdo et al. [34]. All measured values listed were obtained at the baseline examination

*Median (range)

Repeatability of cpRNFLT, MGCIPLT, MGCCT and GCIPLT_4TestPoints_ measurement results calculated using the two measurements obtained at enrollment during two separate sessions [44] were 3.1, 1.1, 1.9 and 1.6 μm, respectively. Aging-associated change rates of cpRNFLT were not significantly different from zero (coefficient for duration or time lapsed was not significantly different from zero), but higher subjects’ baseline age showed significant negative correlation to aging associated change rates of cpRNFLT {-0.013 µm/year/year of age (P=0.031) in an eye with average parametric values of the current cohort (Table 1)} (interaction between duration and baseline age was significant at P=0.029). Baseline thickness, AXL or gender showed no significant effects on the aging associated change rates of cpRNFLT (P > 0.292) (Table 2). On the other hand, both MGCIPLT and MGCCT significantly declined with aging (P=0.020 and 0.017, respectively) and their aging-associated declining rates in an eye with average parametric values of the current cohort (Table 1) were − 0.064 and 0.095 µm/year, respectively. Further, aging-associated decline rates of MGCIPLT and MGCCT were greater along with their baseline thickness, subject’s baseline age and AXL {interaction between duration and baseline thickness, subject’s baseline age or AXL with duration was significant (P < 0.001 – 0.027)} (Tables 3 and 4). Aging-associated changes of the GCIPLT_4TestPoints_ of an eye with average parametric values of this cohort (Table 1) showed similar tendency to that of the MGCIPLT, i.e., the GCIPLT over the whole macular area. Aging-associated decline rates of GCIPLT_4TestPoints_ were not significant (− 0.095 µm/year, P=0.066), but the effect of subjects baseline age and AXL on the aging-associated decline rates of GCIPLT_4TestPoints_ was significant, being close to the value obtained for MGCIPLT (− 0.011 µm/year/year of age, P=0.003, and − 0.098µm/year/mm, P=0.026 versus − 0.009 µm/year/year of age, P < 0.001, and − 0.061µm/year/year of age, P=0.011, respectively) (Table 5).

**Table 2:** Factors contributing to aging-associated change of cpRNFLT

Item	Estimate*(SE), *P* value
Intercept	− 1.879 (5.68), 0.741
Duration=time (years)	0.153 (0.082), 0.062
Thickness at baseline (μm)	0.986 (0.022), < 0.001
Thickness at baseline × duration	− 0.003 (0.008), 0.711
Age at baseline (years)	− 0.011 (0.017), 0.510
Age at baseline × duration	− 0.013 (0.006), 0.031
Axial length (mm)	− 0.065 (0.203), 0.751
Axial length × duration	− 0.076 (0.072), 0.292
Gender (male vs. female)	0.646 (0.430), 0.133
Gender (male vs. female) × duration	0.029 (0.150), 0.849
Image quality score	0.105 (0.019), < 0.001

SE, standard error; cpRNFLT, circumpapillary retinal nerve fiber layer thickness; thickness at baseline × duration, interaction between the thickness at baseline and duration (time lapse from the baseline measurement); age at baseline × duration, interaction between baseline age and duration (time lapse from the baseline measurement); Axial length × duration, interaction between Axial length and duration (time lapse from the baseline measurement); Gender (male vs. female) × duration, interaction between gender area and duration (time lapse from the baseline measurement)

*Estimated coefficient value

**Table 3:** Factors contributing to aging-associated change of MGCIPLT

Item	Estimate* (SE), *P* value
Intercept	3.06 (2.14), 0.153
Duration=time (years)	− 0.064 (0.027), 0.020
Thickness at baseline (μm)	0.979 (0.014), < 0.001
Thickness at baseline × duration	− 0.014 (0.005), 0.009
Age at baseline (years)	− 0.019 (0.006), 0.001
Age at baseline × duration	− 0.009 (0.002), < 0.001
Axial length (mm)	− 0.029 (0.065), 0.662
Axial length × duration	− 0.061 (0.024), 0.011
Gender (male vs. female)	0.071 (0.142), 0.618
Gender (male vs. female) × duration	0.071 (0.051), 0.159
Image quality score	0.001 (0.006), 0.839

SE, standard error; MGCIPLT, the mean of macular ganglion cell-inner plexiform layer thickness; thickness at baseline × duration, interaction between the thickness at baseline and duration (time lapse from the baseline measurement); age at baseline × duration, interaction between baseline age and duration (time lapse from the baseline measurement); Axial length × duration, interaction between Axial length and duration (time lapse from the baseline measurement); Gender (male vs. female) × duration, interaction between gender area and duration (time lapse from the baseline measurement)

*Estimated coefficient value

**Table 4:** Factors contributing to aging-associated changes of MGCCT

Item	Estimate*(SE), *P* value
Intercept	− 5.13 (3.38), 0.128
Duration=time (years)	− 0.095 (0.040), 0.017
Thickness at baseline (μm)	0.995 (0.013), < 0.001
Thickness at baseline × duration	− 0.011 (0.005), 0.027
Age at baseline (years)	− 0.009 (0.008), 0.283
Age at baseline × duration	− 0.013 (0.003), < 0.001
Axial length (mm)	0.132 (0.100), 0.185
Axial length × duration	− 0.089 (0.036), 0.013
Gender (male vs. female)	0.137 (0.188), 0.466
Gender (male vs. female) × duration	0.164 (0.074), 0.026
Image quality score	0.058 (0.009), < 0.001

SE, standard error; MGCCT, the mean of macular ganglion cell complex thicknesses; thickness at baseline × duration, interaction between the thickness at baseline and duration (time lapse from the baseline measurement); age at baseline × duration, interaction between baseline age and duration (time lapse from the baseline measurement); Axial length × duration, interaction between Axial length and duration (time lapse from the baseline measurement); Gender (male vs. female) × duration, interaction between gender area and duration (time lapse from the baseline measurement)

*Estimated coefficient value

**Table 5:** Factors contributing to aging-associated changes of the GCIPLT_4TestPoints_

Item	Estimate* (SE), *P* value
Intercept	6.87 (3.48), 0.048
Duration=time (years)	− 0.095 (0.052), 0.066
Thickness at baseline (μm)	0.987 (0.017), < 0.001
Thickness at baseline × duration	− 0.011 (0.007), 0.103
Age at baseline (years)	− 0.030 (0.009), 0.001
Age at baseline × duration	− 0.011 (0.004), 0.003
Axial length (mm)	0.186 (0.111), 0.094
Axial length × duration	− 0.098 (0.044), 0.026
Gender (male vs. female)	0.057 (0.216), 0.792
Gender (male vs. female) × duration	0.144 (0.097), 0.135
Image quality score	0.006 (0.011), 0.608

SE, standard error; GCIPLT_4TestPoints,_ the mean of ganglion cell-inner plexiform layer (ganglion cell complex) thicknesses in a circular retinal area with a diameter of 0.6 mm (corresponding to about 2 degrees of visual angle) in the four central test points of the HFA 24-2 test, adjusted for RGC displacement according to Drasdo et al. [34] ; thickness at baseline × duration, interaction between the thickness at baseline and duration (time lapse from the baseline measurement); age at baseline × duration, interaction between baseline age and duration (time lapse from the baseline measurement); Axial length × duration, interaction between Axial length and duration (time lapse from the baseline measurement); Gender (male vs. female) × duration, interaction between gender area and duration (time lapse from the baseline measurement). *Estimated coefficient value.

During the 3-year prospective follow-up, ocular transparent media including lens showed no changes on biomicroscopic examination. The mean sensitivity over the whole field (VF_mean_) and that of the four central test points of the HFA 24-2 (VF_4TestPoints_) of an eye with the average parametric values of this cohort (Table 1) showed significantly negative longitudinal time changes (physiological aging-associated declines) of − 35.1 and − 82.1[1/Lambert]/year (*P*<0.001) (corresponding to − 0.15 and − 0.19 dB/year), respectively, significantly more negative by − 2.1 and − 4.0 [1/Lambert]/years with older baseline ages (P < 0.001), and by − 0.10 and − 0.14 [1/Lambert]/[1/Lambert] with higher baseline sensitivities (P < 0.001), respectively. Further, the VF_mean_ and VF_4TestPoints_ measurement results themselves were also significantly lower by − 5.4 and − 13.8 [l/Lambert]/year with older baseline age (P < 0.001), and higher by 0.64 and 0.47 [1/Lambert]/[1/Lambert] with higher baseline sensitivity (P < 0.001), respectively (Tables 6 and 7).

**Table 6:** Factors contributing to aging-associated changes of VF_mean_

Item	Estimate*(SE), *P* value
Intercept	802.3 (457.0), 0.0837
Duration=time (years)	− 35.1 (5.1), < 0.001
VF_mean_ at baseline (1/Lambert)	0.64 (0.06), < 0.001
VF_mean_ at baseline × duration	− 0.10 (0.02), < 0.001
Age at baseline (years)	− 5.43 (0.005), < 0.001
Age at baseline × duration	− 2.07 (0.47), < 0.001
Axial length (mm)	− 1.36 (16.42), 0.934
Axial length × duration	2.05 (5.38), 0.703

SE, standard error; VF_mean,_ mean visual field sensitivity of the all test points of the HFA 24-2 test (1/Lambert); VF_mean_ × duration, interaction between VF_mean_ at baseline and duration (time lapse from the baseline measurement); age at baseline × duration, interaction between baseline age and duration (time lapse from the baseline measurement); Axial length × duration, interaction between Axial length and duration (time lapse from the baseline measurement)

*Estimated coefficient value

**Table 7:** Factors contributing to aging-associated changes of VF_4TestPoints_

Item	Estimate*(SE), *P* value
Intercept	2610.1 (994.5), 0.011
Duration=time (years)	− 82.1 (13.5), <0.001
VF_4TestPoints_ at baseline (1/Lambert)	0.47 (0.07), <0.001
VF_4TestPoints_ at baseline × duration	-0.14 (0.03), < 0.001
Age at baseline (years)	− 13.8 (2.90), < 0.001
Age at baseline × duration	− 4.03 (1.21), < 0.001
Axial length (mm)	− 28.1 (35.1), 0.426
Axial length × duration	− 4.80 (14.7), 0.744

SE, standard error; VF_4TestPoints,_ mean visual field sensitivity of the central four test points of the HFA 24-2 test (1/Lambert); VF_4TestPoints_ at baseline × duration, interaction between VF_4TestPoints_ at baseline and duration (time lapse from the baseline measurement); age at baseline × duration, interaction between baseline age and duration (time lapse from the baseline measurement); Axial length × duration, interaction between Axial length and duration (time lapse from the baseline measurement)

*Estimated coefficient value

## Discussion

In the current study, the longitudinal aging-associated changes in the SD-OCT-measured thicknesses of the RGC-related retinal layers (RGC-RRLT) were studied in normal Japanese subjects with an average age of 50 years. Because of well-known ethnic differences in the RGC-RRLT measurement results in normal subjects [14, 15, 21], and dependence of the measurement results on the SD-OCT instruments used [23–27], it is of primary importance to provide aging-associated longitudinal change rates of cpRNFLT, MGCIPLT or MGCCT in healthy eyes for each ethnicity and record the SD-OCT instrument used to correctly estimate glaucoma-caused longitudinal changes of these important parameters.

Using Cirrus HD-OCT (Carl Zeiss Meditec), aging associated decline rates from − 0.16 to − 0.52 μm/year and of − 0.32 μm/year are reported for cpRNFLT [17, 20, 22] and for MGCIPLT [18], respectively, in normal subjects of mainly European descent. Using Spectralis OCT (Heidelberg Engineering GmbH), aging associated decline rates of − 0.44μm/year are reported for cpRNFLT in persons of European descent [19, 21] and of − 0.51μm/year in persons of African descent [21] all of them healthy subjects. In the current study where aging-associated change rates of cpRNFLT, MGCIPLT and MGCCT were measured using 3D-OCT 2000 (Topcon) every 3 months for 3 years in 37 normal Japanese subjects (mean age, 50.4 years), For cpRNFLT, aging-associated decline could not be detected, while MGCIPLT and MGCCT showed significant aging-associated decline of − 0.064 and − 0.095 μm/year, respectively. The findings obtained for cpRNFLT were unexpected, since VF sensitivity should approximately correspond to the retinal area covered by cpRNFLT in the same eye; the mean sensitivity over the HFA 24-2 VF, VF_mean_, showed significant aging-associated decline rate of − 35.1[1/Lambert)]/year, corresponding to − 0.15 dB/year which agreed with that reported by a cross-sectional and longitudinal study in normal Japanese [10]. This reasonable result obtained for VF sensitivity in the same eyes suggests that the current subjects were not significantly biased from the general normal population.

Larger test-retest variability, fewer measurement points, and a shorter follow-up period should reduce the power to detect a significant trend. Inter-visit test-retest variabilities of the cpRNFLT, MGCIPLT, MGCCT and GCIPLT_4TestPoints_ with the current instruments were 3.1 μm, for cpRNFLT and 1.1 μm, 1.9 μm and 1.6 μm for MGCIPLT, MGCCT and GCIPLT_4TestPoints_, respectively, favorably compared with those reported in the literature for measurement results with various SD-OCT instruments [44, 45]. The follow-up period and intervals in the current study were similar to (3 years vs. 1.7–4.5 years and 3 months vs. 3.5–6 months, respectively) those adopted by previous studies [17–20]. Thus, it seems unlikely that a shorter follow-up period or fewer measurements mainly accounted for an undetected significant trend in longitudinal decline in the cpRNFLT in the current subjects. The current study indicates that in older subjects, the aging-associated decline rates in the cpRNFLT were found to be significantly more negative (interaction between the subject’s baseline age and duration were significant) by about − 0.01 μm/year/year of age, suggesting that in the cohort older than the current ones with mean age of 50.4 years, the aging-associated decline rates of the cpRNFLT might become sufficiently negative to be detected. The fact that the same analysis, when applied to the older group of the current subjects aged 50.4 years or older did not yield a significantly negative aging-associated decline rates does not necessarily contradict with the above speculation, since this stratified analysis included only 39 eyes of 20 subjects, hence half the number of subject eyes and the same number of explanatory variables should have considerably reduced the statistical power of detection (Supplementary Table 1). Somewhat younger ages of subjects in the current study than in the previous studies (50 vs. 56–65 years) [17–22] may be at least partly responsible for the discrepancy between the current and their results. Rates of aging-associated declines in the cpRNFLT were reportedly less than predicted by aging-associated decline in the number of RGCs, attributed to the presence of differential aging-associated declines of the non-neuronal components in the RNFL [46, 47]. An ethnic difference in the cpRNFLT has been reported [14, 15] and a comparison of the Bruch membrane’s opening-minimumu rim width and cpRNFLT between normal Japanese and Caucasians subjects suggests a difference in the ratio of the amount of RGC axons to that of the non-neuronal components in the RNFL between them [48]. Provided there are ethnic differences in the amount of physiological aging-associated changes in the non-neuronal component between Japanese subjects and other groups, this might partly explain the fact that aging-associated cpRNFLT decline could be detected in European subjects [17–22], but not in the current Japanese subjects. On the other hand, aging-associated decline rates of MGCIPLT and MGCCT were significant averaging − 0.064 μm and − 0.095μm/year, respectively. However, these rates also seemed to be considerably smaller than − 0.32 μm/year reported in normal subjects of mainly European descent [18], suggesting possibility that there is an ethnic difference in aging-associated decline rates of RGC-RRLTs and rates were smaller in Japanese than in European descent subjects. The fact that a small decline rate could be detected for MGCIPLT or MGCCT, but not for cpRNFLT, could be also compatible with the lower measurement repeatability of cpRNFLT than that of MGCIPLT or MGCCT (3.1 μm vs 1.1 μm or 1.9 μm). For cpRNFLT, MGCIPLT and MGCCT, aging-associated change rates were found to be significantly more negative along with thicker baseline thickness and higher subjects’ baseline age. Further, for MGCIPLT and MGCCT, rates were significantly more negative along with longer AXL or higher myopic power. Positive correlation of decline rates with thicker baseline thickness is understandable, given that a same aging-associated loss rate of each RGC, a greater number of baseline RGCs (∝ thicker baseline cpRNFLT, MGCIPLT or MGCCT) will result in a greater number of RGCs yearly lost (∝ yearly decline rate of cpRNFLT, MGCIPLT or MGCCT). Positive correlation of subjects’ age and AXL with MGCIPLT and/or MGCCT decline rate is interesting, but understandable, since both subjects’ age and myopia [49, 50] are well known risk factors for development of open angle glaucoma.

In the current study, the longitudinal aging-associated changes in the VF sensitivity and SD-OCT-measured RGC-RRLT in the corresponding area were measured in the same eyes of normal Japanese subjects. The correspondence should be more exact between the mean sensitivity of the central 4 test points of HFA 24-2, VF_4TestPoints_ and GCIPLT_4TestPoints_ than between the mean sensitivity over the HFA 24-2 VF, VF_mean_, and cpRNFLT. So, it may be interesting to compare the corresponding VF_4TestPoints_ and GCIPLT_4TestPoints_. Although GCIPLT_4TestPoints_ decline rate was not statistically significant (P=0.066), it should be greater than of MGCIPLT of -0.064 μm/year which was statistically significant, since GCIPLT_4TestPoints_ was thicker than MGCIPLT and decline rates of MGCIPLT were significantly greater as baseline thickness increased (interaction between baseline thickness and duration was significant). Further, similar effects of baseline age and AXL on the thickness decline rate were seen for both MGCIPLT and GCIPLT_4TestPoints_. So, GCIPLT_4TestPoints_ decline rate of − 0.095 μm/year is thought to be not far from the reality. The decline rate of VF linear sensitivity of -82.1[1/Lambert]/year (− 0.19 dB/year) in VF_4TestPoints_ corresponded to that of − 0.095 μm/year in GCIPLT_4TestPoints_.

Using cross-sectional data of normal Japanese with the mean age of 49 years, we previously estimated that aging associated decline rate inVF_4TestPoints_ of 1000/[1/Lambert]/decade roughly corresponds to that of a decline in GCIPLT_4TestPoints_ of 1.6 μm/decade [16]. If, based on the current results of a yearly decline rate in VF_4TestPoints_ and GCIPLT_4TestPoints_, we estimate the yearly decline rate of GCIPLT_4TestPoints_ against a yearly decline rate of VF_4TestPoints_ of 1000[1/Lambert]/decade, calculated to be 1.2 μm/decade, there will be a reasonable agreement with the value of 1.6 μm/decade, provided we take the difference in the cross-sectional and longitudinal analysis results into consideration.

Not only aging-associated changes in the retinal neurons but also in the neurons in the central visual pathway are related to a physiologic aging-associated decline in the perceived VF sensitivity in a specific retinal area. Since the cpRNFLT is related to the number of RGC axons and non-neuronal cells in the peripapillary retina, but not to neurons in the central visual pathway, the physiologic aging processes at age 50 years might be reflected with more sensitivity in the perceived VF sensitivity decline than in the OCT-measured cpRNFLT, which may partly be attributable to the fact that a significant physiological aging-associated decline in the perceived VF sensitivity is associated with insignificant physiological aging-associated decline in the OCT-measured cpRNFLT revealed by the present study. It is possible that the relationship between the OCT-measured structure and SAP-measured function (VF sensitivity) is not the same between the physiological aging process [16] and glaucoma-caused damaging process [51]. Glaucomatous damage primarily affects the RGCs and their axons and secondarily the neurons in the central visual pathway. In this case, the perceived SAP sensitivity decline may have been dampened by the plasticity of the visual cortex and normal cerebral adaptation to chronic deterioration in the visual input caused by slowly progressing glaucomatous RGC damage [52].

The current results have clinical implications in managing Japanese glaucoma patients using the SD-OCT 2000 (Topcon), which is widely used in Japan. To evaluate glaucoma-caused structural deterioration rates of MGCIPLT or MGCCT in patients with mean age of 50 years, aging-associated decline rates of MGCIPLT and MGCCT of − 0.064 and − 0.095 μm/year must be discounted. Further, for correct inter-group comparisons of cpRNFLT, MGCIPLT or MGCCT, adjustments must be made not only for subjects’ age, but also for AXL must be adjusted for correct inter-group comparison of MGCIPLT and MGCCT. A small time-dependent change of cpRNFLT might not be sensitively detected using SD-OCT 2000 in normal Japanese eyes aged between 40 and 60 years. If it could be detected, the detected change might be mainly attributable to a disease.

The current study has limitations. First, the number of normal subjects was small and the follow-up period may not have been sufficiently long, resulting in a lower statistical capability to detect significant time changes in the RGC-related retinal layer thickness measured using a current SD-OCT instrument. As discussed previously, however, the cpRNFLT or GCIPLT time change rates of about 0.3–0.5 μm/year are reported in previous studies with a similar number of normal subjects and follow-up periods [17–21], and the test-retest variation of the current SD-OCT 2000 measurement results was thought to be reasonably satisfactory compared with those reported for other SD-OCT instruments [44, 45]. These results suggest that the statistical power to detect the time change rates of the CpRNFLT in the current normal subjects should be comparable to those of previous studies that detected significant aging-related declines of the cpRNFLT [17–21]. The mean age of the current normal Japanese subjects was 50 years and, as discussed previously, the physiologic aging-related decline of the RGC-RRLTs would be greater in normal Japanese subjects with a higher age. Thus, it should be noted that the current results were applicable to Japanese subjects aged around 50 years. Finally, the current results were obtained using Topcon SD-OCT 2000 in normal Japanese. Since there exist reported ethnic differences in the SD-OCT-measured RGC-related retinal layers [14, 15, 21] and the thickness measurement results obtained with different SD-OCT instruments, these are not necessarily interchangeable [23–27], it must be noted that the current results can be used as a reference point to those obtained with Topcon SD-OCT in Japanese.

### Supplementary Information

Below is the link to the electronic supplementary material.

Supplementary file1 (DOCX 20 KB)
