# Supplementary material for: Aging-associated changes of optical coherence tomography-measured ganglion cell-related retinal layer thickness and visual sensitivity in normal Japanese
Source: Jpn J Ophthalmol. 2024 Mar 18;68(2):117–25. doi: 10.1007/s10384-024-01049-3 (PMC10963440; doi:10.1007/s10384-024-01049-3)
Supplement: Supplementary file 1 — Supplementary file1 (DOCX 20 KB) [file 10384_2024_1049_MOESM1_ESM.docx]

**Supplement.**

Taking one of the central 4 test points of HFA 24-2 (C1) as an example, below is a description of how the location of the retinal area corresponding to C1 and the location of a point on the fundus camera film plane (OCT image sensor plane) corresponding to that retinal area were identified.

1) A ray from C1 which can be considered a paraxial ray with sufficient precision undergoes refraction while bending through the optical system of the subject eye (Eye1) and then reaches the retina. Based on the anterior corneal radius, axial length (AXL) and total refractive error of Eye1 which were measured, some of the parameters of Gullstrand schematic eye (GSE) were modified to make the GSE optically equivalent to Eye1 (GSE-Eye1). To be specific, Eye1’s anterior corneal radius and AXL are adopted as those of GSE-Eye1, and lens thickness, anterior curvature radius and posterior curvature radius of the lens of GSE were changed step by step to make total refractive error of GSE-Eye1 equal to that of Eye1. Using GSE-Eye1, the location on the fundus of GSE-Eye1 which the ray from C1 reaches is readily calculated using paraxial ray-tracing algorithm. Let it be assumed that the distance between that location and visual axis center of GSE-Eye1’s fundus is X. It is known that the location of photoreceptors and corresponding retinal ganglion cells is somewhat different within the 10 degrees of central visual field. The location of the retinal ganglion cells corresponding to X can be calculated based on the formula of Drasdo et al. (Ref. 34) (X_D_). X_D_ is uniquely determined as a function of the cornea radius, axial length (AXL) and total refractive error of Eye1 and location information of C1.

2) Using the detailed optical properties of the OCT camera system adjusted according to the total refractive error of Eye1 (=GSE-Eye1) to focus on the fundus of GSE-Eye1 and GSE-Eye1, a new optical system consisting of the OCT-camera and GSE-Eye1 is constructed. By applying paraxial ray-tracing algorithm to this system, the location of a point on the OCT-image sensor which corresponds to the point with a distance from the visual axis center on the GSE-Eye1 fundus of X_D_ is calculated. Let the distance between this point on the OCT image sensor plane and visual axis center (center of fovea) of the OCT image censor be Y_D_.

Y_D_/X_D_ is usually different from 1.0, depending on the optical properties of each subject eye and fundus camera, and the Y_D_/X_D_ value is designated as a magnification correction factor for each subject eye. The procedure to calculate Y_D_/X_D_ value for each subject eye is called magnification correction. In the current study, Y_D_ is directly calculated using paraxial ray-tracing algorithm on a personal computer and to know the Y_D_/X_D_ value for each subject eye is not considered important.

**Supplemental Table 1. Factors contributing to aging-associated change of CpRNFLT in younger and older subjects’ cohort divided by the mean age of the cohort**

| **Item** | **Estimate*(SE), *P* value** | |
| --- | --- | --- |
| Age stratification (years) (eyes/subjects) | ≤50.4 (34/17) | >50.4 (39/20) |
| Intercept | -1.864 (7.346), 0.800 | 0.899 (8.872), 0.919 |
| Duration=time (years) | -0.328 (0.230), 0.154 | -0.038 (0.205), 0.852 |
| Thickness at baseline (μm) | 0.970 (0.034), <0.001 | 0.993 (0.034), <0.001 |
| Thickness at baseline × duration | 0.019 (0.015), 0.219 | -0.014 (0.012), 0.253 |
| Age at baseline (years) | -0.015 (0.031), 0.622 | -0.019 (0.050), 0.706 |
| Age at baseline × duration | -0.040 (0.014), 0.004 | 0.007 (0.016), 0.676 |
| Axial length (mm) | 0.056 (0.215), 0.794 | -0.216 (0.346), 0.532 |
| Axial length ×duration | -0.029 (0.095), 0.757 | -0.015 (0.119), 0.896 |
| Gender (male vs. female) | -0.195 (0.492), 0.693 | 1.231 (0.617), 0.046 |
| Gender (male vs. female) x duration | 0.160 (0.218), 0.465 | 0.018 (0.203) 0.928 |
| Image quality score | 0.088 (0.025), <0.001 | 0.113 (0.028), <0.001 |

SE, standard error; CpRNFLT, circumpapillary retinal nerve fiber layer thickness; thickness at baseline × duration, interaction between the thickness at baseline and duration (time lapse from the baseline measurement); age at baseline × duration, interaction between baseline age and duration (time lapse from the baseline measurement); Axial length × duration, interaction between Axial length and duration (time lapse from the baseline measurement); Gender (male vs. female) × duration, interaction between gender area and duration (time lapse from the baseline measurement). *Estimated coefficient value.
